# Using Google Search Ads to Promote Lethal Means Safety in Military Veterans: A Mixed-Methods Evaluation of the Keep It Secure Paid Media Campaign

**DOI:** 10.1027/0227-5910/a001057

**Published:** 2026-03-26

**Authors:** Alan R. Teo, Sean P. M. Rice, Summer Newell

**Affiliations:** ^1^ VA Portland Health Care System, Center to Improve Veteran Involvement in Care (CIVIC), Portland, OR, USA; ^2^ Department of Psychiatry, Oregon Health & Science University, Portland, OR, USA; ^3^ School of Public Health, Oregon Health & Science University – Portland State University, Portland, OR, USA

**Keywords:** firearm storage, public health campaign, Department of Veterans Affairs, gun safety, Google keyword search ad

## Abstract

**Abstract:** *Background:* Despite increasing use of Google Ads in public health campaigns, few studies have described their use in suicide prevention and/or examined application to lethal means safety (LMS), a priority for suicide prevention efforts in military veterans. *Aims:* The objective of this study was to evaluate the effectiveness of an LMS campaign for US veterans by analyzing engagement rates across different types of Google Ad search terms included in the campaign. *Methods:* We conducted a secondary data analysis of Google Ads from July 2022 and May 2023 in Keep It Secure, the Department of Veterans Affairs (VA) first national LMS campaign. Our primary outcome was click-through rate (CTR). Our predictor variable was category of Google search. Categories were identified from the user queries employing a team-based immersion/crystallization process, an inductive, iterative qualitative approach. We then used generalized mixed modeling to compare CTR across search categories. *Results:* Using Google Ads, the campaign resulted in a total of 1,849,077 impressions and 342,747 clicks to the campaign website, for an overall 18.5% CTR of 59,587 unique searches, and we categorized 47,387 into 10 categories (Firearm Storage, General VA Inquiries, Veteran Resources, Suicide Prevention, Mental Health, Crisis Line, Suicidal Ideation, Safety Class, Firearm Policy, and Homelessness). Campaign engagement varied significantly across categories (*p* < .001). Searches related to general VA inquiries (30.0% CTR) and veteran resources (23.0% CTR) demonstrated the highest engagement, whereas firearm storage (4.6% CTR) and suicidal ideation (1.3% CTR) were the lowest. *Limitations:* Since this study was a secondary analysis, causal inferences cannot be made. *Conclusions:* Google Ads resulted in high overall engagement in an LMS educational campaign. For LMS outreach to veterans who are not in acute crisis, a “side-door” approach targeting people searching for general VA information and veteran benefits and resources may be more effective than using firearm or suicide-related keywords.

Suicide prevention remains a pressing public health concern, and military veterans constitute a key target population for prevention efforts in the United States. Veterans are more likely to die by suicide than nonveterans (US Department of Veterans Affairs Office of Mental Health and Suicide Prevention, 2023), and they frequently own firearms (Cleveland et al., 2017; Nichter et al., 2024), have experience handling firearms (Anestis & Capron, 2018), and are more likely to die by suicide using firearms (Hoffmire & Bossarte, 2014; Ramchand & Montoya, 2025; US Department of Veterans Affairs Office of Mental Health and Suicide Prevention, 2023) than the general population.

Lethal means safety refers to measures that reduce the ready availability of potentially lethal means to individuals experiencing a suicidal crisis. A range of international studies have found that restricting access to firearms reduces rates of suicide. For example, after military reforms in Switzerland reduced access to firearms in 2003, the overall suicide rate and the firearm suicide rate both declined in intervening years (Reisch et al., 2013). Similarly, after policy change in the Israeli Defense Forces, the suicide rate decreased a staggering 40%, with much of this decline thought to be driven by soldiers becoming less likely to take firearms home over the weekend and thereby reducing household access to firearms (Lubin et al., 2010).

In recent years in the United States, the Department of Veterans Affairs (VA) has focused attention on efforts to increase safe and secure storage of firearms. VA is the largest integrated healthcare system in the United States. It employs over 371,000 staff who serve nine million military veterans through 1,380 facilities, including both larger medical centers and smaller outpatient clinics. VA’s focus on secure storage of firearms has been motivated in part by evidence that suicidal ideation and intent are often fleeting, and increasing the amount of time needed to access a firearm can improve chances that an impulse will pass before a person has the opportunity to act upon it, resulting in a greater chance of survival (Anestis et al., 2020; Shenassa, 2004).

In addition to lethal means safety, another focal point of VA’s suicide prevention strategy has been the implementation of communication campaigns to spread public awareness of and education about suicide. While little information is known about veteran-specific suicide-related internet use, generally research shows that individuals who are suicidal go online for suicide-related information and suicide-related internet use is related to increased past-year suicidal ideation and future suicide risk (Mok et al., 2015; Niederkrotenthaler et al., 2017). Furthermore, the first recommendation of the PREVENTS Roadmap, created in response to Executive Order (EO) 13,861 signed by President Trump in 2019, was to “Create and implement a national public health campaign focused on suicide prevention for Veterans and all Americans” (Executive Order No. 13,861, 2019, p. 18).

Results from systematic reviews and other studies suggest that public health campaigns can be an effective suicide prevention tactic. For instance, suicide prevention campaigns can increase suicide knowledge and awareness, positively changing attitudes, and increase help-seeking (Pirkis et al., 2019; Torok et al., 2017). Successful campaigns are thought to contain a solid theoretical basis, use a multilevel approach, be tailored to a specific population, and be thoughtfully disseminated with community engagement (Calear et al., 2014; Dumesnil & Verger, 2009; Pirkis et al., 2019; Torok et al., 2017).

One component of advertising that may be particularly relevant to public health campaigns is Google Ads. Google Ads represent a form of paid media – meaning advertising that involves paying for its placement. Google Ads is an online platform where advertisers can pay to place a sponsored link to a website in front of individuals searching on Google for relevant topics. These ads frequently appear at or near the top of search results and can contain campaign messages accompanied with a website link. Google Ads are particularly appealing since Google has, by far, the largest search engine market share (91.1%; StatCounter, n.d.), and an average of 40,000 searches are made on Google every second (Internet Live Stats, n.d.).

While literature on the application of Google Ads to medical research and mental health campaigns is still relatively new, a handful of studies have suggested potential applications (Murphy et al., 2018; Onie et al., 2023, 2024; Upadhyay et al., 2020). For instance, a 2018 study evaluated a Canadian initiative for men with mental health or substance use conditions found that website visits, but not visit duration or returning users, increased with use of Google Ads (Murphy et al., 2018). A 2020 US study found Google Ads useful to recruit pregnant women considering abortion into a research study, noting 69% retention rate at one-month with participants recruited this way (Upadhyay et al., 2020). Most recently, two studies by the same research team have examined Google Ad campaigns for suicide prevention (Onie et al., 2023, 2024). In the first study in the United States, Google Ads led to 664 clicks in the first 16-day phase of the study, then nearly 10-fold more clicks in the second 19-day phase. The ads were found to be effective in rapidly generating engagement above and beyond the presence of a suicide hotline banner at the top of the search webpage (Onie et al., 2023). In a subsequent study conducted in Australia, Google ads were varied in terms of whether they included explicit wording about suicide. Results showed that engagement in the campaign depended on whether individuals were searching using low-risk (e.g., “feeling so alone”) versus high-risk (e.g., “I want to die”) keywords (Onie et al., 2024). We are not aware of any empirical studies that have examined Google Ads in the context of promoting lethal means safety. The aims of our project were to characterize the types of Google searches included in the Keep It Secure campaign and determine their association with campaign engagement.

## Materials and Methods

### Data Source and Data Collection

This study was designed as a secondary data analysis of the Keep It Secure campaign, VA’s first national public health campaign specifically designed to address lethal means safety among veterans (US Department of Veterans Affairs, 2018). We have previously published on development of the Keep It Secure campaign (Teo et al., 2024). Briefly, campaign goals were to: (1) increase awareness and understanding that locking firearms can reduce suicide, and (2) encourage adoption of secure storage of firearms. The campaign was executed in distinct phases, called flights. This study analyzes data collected during Flights 3 and 4, which occurred between July 2022 and May 2023. While earlier phases relied primarily on public service announcement videos, the timeframe for this study marked the introduction of a responsive keyword search component.

**Figure 1:**
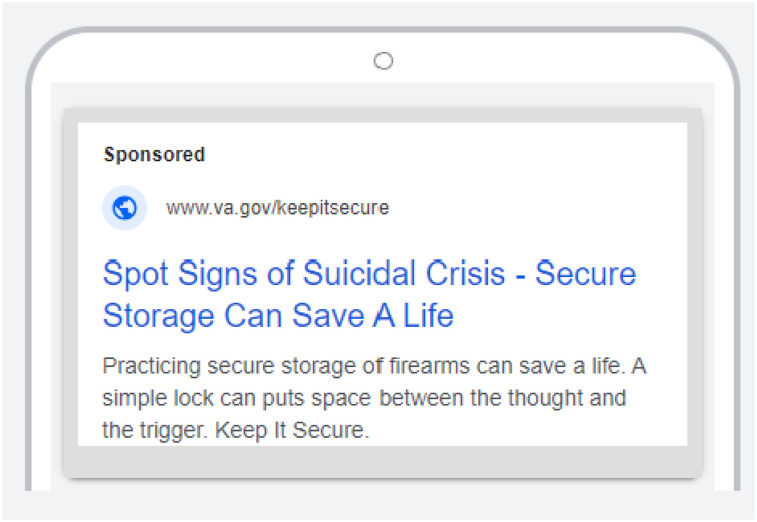
Example of a possible Google Keyword Search Ad for the Keep It Secure Campaign.

Ad creation and function were as follows:

• Ad generation: A marketing company contracted with VA used focus groups comprised of veterans and their families to help craft ad messaging. Ultimately, messaging centered on the concept that “a simple lock puts space between the thought and the trigger,” framing secure storage as a safety measure rather than a restriction on gun ownership.
• Ad description and presentation: Ads were presented to Google users via a responsive keyword search campaign, which is designed to capture user intent at the moment of information seeking. This meant that ads were not static; instead, Google’s proprietary algorithm and platform selected from ad components provided by the marketing team (e.g., headlines, descriptions, and images). An example of a potential ad is presented in Figure 1.
• Triggering mechanism: Ads appeared at the top of the results page when a user’s query matched campaign-relevant keywords related to firearm safety, storage, suicide prevention, etc. Keywords are described further below in the results. Upon clicking an ad, the user was redirected to the campaign website, which hosted educational resources, downloadable information sheets, and links to crisis support. (The campaign website site has been modified since the time of the campaign but the current version can be seen at KeepItSecure.net)

In terms of ethics approval, this project was determined by the Institutional Review Board (IRB) at the VA Portland Health Care System not to be human subjects research (i.e., exempt from IRB review) on May 5, 2022. The data that support the findings of this study are available from the corresponding author upon reasonable request.

### Statistical Analyses

#### Qualitative Analyses

We first aggregated raw ad data to the search-level, such that each unique Google search (i.e., the exact text of the search did not match any other search) had an associated number of impressions (i.e., ad views) and clicks to the Keep It Secure website. Employing the immersion/crystallization method (Borkan, 1999, 2022), an inductive and iterative qualitative analysis approach, the experienced analyst team (SN, SR) engaged in multiple rounds of reviewing the unique Google searches, leading to identification of meaningful categories. Once a potential category was identified (e.g., Firearm Storage), we used keyword searches to identify queries related to firearm storage (e.g., “gun safes”). See Table 1 for more details. Both analysts then verified that the queries captured in the new category were accurate and made corrections based on ongoing debriefing between analysts in all identified categories. All analytical decisions were made collectively between the analyst team, employing peer review and debriefing, as well as prolonged engagement and persistent observation in all stages of the analysis (Morse, 2015). We continued to review uncategorized data to identify new categories until reaching pattern saturation. In some cases, the team collectively decided to collapse, or divide into new, categories – part of the immersion/crystallization process. We next removed searches from the categorized dataset if they fell into multiple categories. This initial process resulted in categorization of 46,682 of 59,587 unique Google searches.

**Table 1:** Descriptive statistics and overall summaries of qualitatively derived search categories

Category	Description	Example searches	Searches (total = 59,587)	Impressions (total = 1,849,077)	Clicks (total = 342,747)	Click-through rate (overall = 18.5%)
Firearm storage	Related to devices that keep firearms secure, most commonly a firearm safe, cabinet, or lock	Affordable gun safes, bed gun safe, biometric pistol safe, oak gun cabinet	16,183 (27.2%)	252,117 (13.6%)	11,494 (3.4%)	4.6
General VA inquiries	VA website, clinic locations, My HealtheVet, etc.	Baltimore VA office, call veterans administration, closest VA clinic	10,825 (18.2%)	752,526 (40.7%)	226,109 (66.0%)	30.0
Veteran resources	Primarily inquiries regarding eligibility for VA and other veteran-based services. Also includes veteran-focused organizations, foundations, charities, and veteran service organizations	Am I eligible for VA benefits, DAV Cincinnati, disability benefits VA, eligibility for VA, free legal help for veterans, red cross assistance for veterans	4,605 (7.7%)	246,571 (13.3%)	56,641 (16.5%)	23.0
Suicide prevention	Prevention of suicide or firearm-related injuries Also includes suicide prevention statistics, methods, safety planning, suicide risk screening, and awareness.	Suicide awareness, VA suicide prevention, 22 veterans a day	3,739 (6.3%)	116,619 (6.3%)	5,783 (1.7%)	5.0
Mental health	General mental health questions, mental health diagnoses (e.g., PTSD, depression, anxiety), and treatment	Air force mental health services, art therapy for veterans, counseling PTSD veterans, depression in army, facts about veterans mental health	3,520 (5.9%)	85,486 (4.6%)	6,898 (2.0%)	8.1
Crisis line	Any crisis line, including the veterans crisis line. Also includes searches for chats or texts with crisis counselors	24 h crisis, 988 helpline, veterans crisis line, army crisis hotline, can you text a suicide hotline	2,635 (4.4%)	211,683 (11.4%)	26,842 (7.8%)	12.7
Suicidal ideation	Inquiries along the lines of “kill myself” or “suicidal thoughts”	Is it normal to want to die, kill yourself now, reasons not to die, signs someone is going to kill themselves, suicidal thoughts help	2,535 (4.3%)	53,864 (2.9%)	710 (0.2%)	1.3
Safety class	Inquiries related to firearm safety classes, or the permitting process to own firearms or concealed carry	Colorado hand gun safety, conceal and carry license, firearm safety certificate	1,801 (3.0%)	17,048 (0.9%)	1,200 (0.4%)	7.0
Firearm policy	Inquiries related to firearms ownership politics, restrictions, and laws and bills	Arguments for and against gun control, California gun rights, current firearm legislation, gun control bill, gun laws	1,122 (1.9%)	8,278 (0.4%)	510 (0.1%)	6.2
Homelessness	Housing needs and homelessness among veterans	Emergency housing for veterans, ending homelessness among veterans, how to help homeless veterans	422 (0.7%)	14,742 (0.8%)	2,361 (0.7%)	16.0

Finally, we sorted the uncategorized data by descending number of impressions, and the qualitative analyst (SN) manually coded all searches with at least 50 impressions, which added an additional 705 unique searches for a total of 47,387 unique searches. Because there are no scientifically validated cutoffs for impressions using Google Ads, and previous research has found no correlation between impressions and click-through rate using other platforms (Nayak et al., 2023), we opted to limit analysis to 50+ impression searches to target the searches made most often during the campaign period. Certain searches were deemed uncategorizable (e.g., “gunclass mudbaby”) and remained uncategorized. See Figure 1 in Electronic Supplementary Material 1 (ESM 1) for additional details on the analytic decision-making process.

#### Quantitative Analyses

Once we assigned categories to searches, we calculated the click-through rate (CTR) for each unique search (excluding noncategorized searches). To account for instances where a single impression generated multiple clicks, any resulting click-through rates (CTRs) exceeding 100% were capped at a maximum value of 100% to maintain a proportional distribution of 0 to 100%. Due to the extreme skewness and kurtosis, the percentage nature of the data, large zero-inflation, and presence but not inflation of 1s in the distribution (precluding quantile and zero-one inflated beta regression), we created two CTR variables for analysis: CTR0 (i.e., a binary variable indicating a search having either 0% CTR or > 0% CTR) and CTR+ (i.e., a continuous CTR value when raw CTR was above 0). We then applied two sets of generalized mixed models (GMMs). First, we compared average CTR + per search across categories using a GMM with Gaussian distribution, identity link, and Laplace estimation. We set significance of the Wald F-test a priori at *p* < .05, and least square means were compared pairwise using Bonferroni-corrected *p*-values. We also compared CTR0 using a GMM with binary distribution, logit link, and Laplace estimation. We again used the Wald F-test to test significance of the overall difference between categories (*p* < .05). We then computed pairwise odds ratios (transformed least squares means), inferring significance when the 95% confidence interval did not contain 1 after Bonferroni correction.

## Results

### Descriptive Characteristics

Our dataset included campaign ads from 275 nonconsecutive days between July 2022 and May 2023. Google Ads were associated with 1,849,077 impressions and 342,747 clicks to the campaign website, for an overall click-through rate (CTR) of 18.5%. A total of 248,604 unique words were represented across all searches. Of the 59,587 unique searches, 39,253 (65.9%) occurred multiple times (range = 2–167,861). About 74.5% of searches resulted in no clicks. Overall, we applied qualitative analysis to 47,387 unique searches (79.5%); 1,758,935 impressions (95.1%), and 338,548 clicks (98.7%). We used these categorized data in our quantitative analyses below.

### Qualitative Analysis and Categorization of Google Searches

Our qualitative analysis resulted in 10 categories of searches: General VA Inquiries, Veteran Resources, Homelessness, Crisis Line, Mental Health, Firearm Policy, Suicide Prevention, Suicidal Ideation, Firearm Storage, and Safety Class. Table 1 provides definitions of each of the categories, example searches that were categorized, and their overall descriptive statistics. General VA inquiries reflected nonspecific Veterans Affairs topics. Veteran Resources reflected benefits/programs to help veterans. Homelessness reflected housing needs and issues. Crisis Line reflected searches related to any help line or chat. Mental Health reflected mental health information, therapies, and diagnoses. Firearm Policy reflected laws or legal proposals surrounding firearms. Suicide Prevention reflected prevention of suicide or firearm-related injuries. Suicidal Ideation reflected thoughts about dying by suicide. Firearm Storage reflected devices or methods to keep firearms secure. Safety Class reflected courses on safe firearm practices and permitting. Search categories also tended to exist in three overarching domains: firearm searches (Firearm Storage, Firearm Policy, Safety Class), mental health searches (Mental Health, Crisis Line, Suicide Prevention, Suicidal Ideation), and general Veteran searches (General VA Inquiries, Veteran Resources, Homelessness).

As shown in Table 1, the largest proportion of unique searches were related to Firearm Storage (27.2%) and General VA Inquiry (18.2%). In terms of proportion of impressions, General VA Inquiries comprised, by far, the largest proportion (40.7%), followed by Firearm Storage (13.6%) and Veteran Resources (13.3%). Homelessness and Firearm Policy contained the smallest proportion of both unique searches (0.7% and 1.9%, respectively) and impressions (0.8% and 0.4%, respectively).

General VA Inquiries, Veteran Resources, and Homelessness had the highest CTRs (30.0%, 23.0%, and 16.0%, respectively), while searches related to Suicidal Ideation, Firearm Storage, and Suicide Prevention had the lowest CTRs (1.3%, 4.6%, and 5.0%, respectively). At the domain level, general Veteran Inquiry searches had the highest CTR (28.1%) compared to mental health (8.6%) and firearm (4.8%) searches.

### Quantitative Analyses Comparing Engagement Across Search Categories

**Figure 2:**
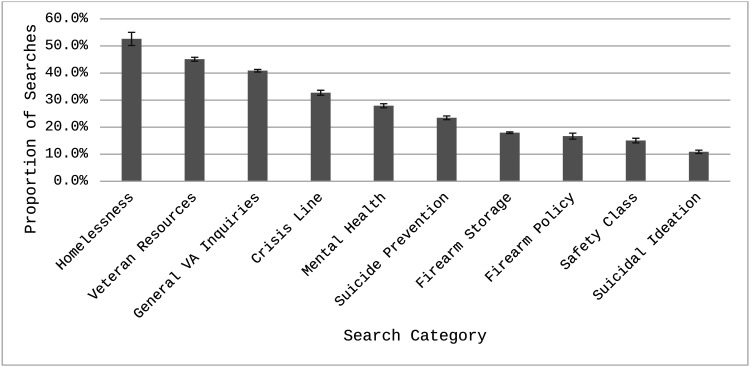
Estimated proportion of searches with at least one click. Proportions are derived from the generalized mixed model predicting an instance of at least one click. Error bars represent standard errors.

Including only categorized searches (*N* = 47,387), the average CTR was 6.9% (95% CI [6.7, 7.1]) per search. Overall, 27.6% of searches were associated with at least one click. There was a significant difference between categories in the likelihood of an ad getting clicked on, *F*(9, 47,377) = 331.5, *p* < .001. Figure 2 presents the proportions of searches with at least one click. The Homelessness category had the largest proportion of searches with at least one click (52.6%, 95% CI [47.8%, 57.3%]), whereas searches in the Suicidal Ideation category were the lowest (10.9%, 95% CI [9.7%, 12.2%]). Firearm Storage (18.0%, 95% CI [17.4%, 18.6%]), Firearm Policy (16.7%, 95% CI 14.6%, 19.0%]), and Safety Class (15.1%, 95% CI [13.5%, 16.8%]) were also low categories in terms of proportion of searches with at least one click. Of the 45 pairwise comparisons, 41 were statistically significant, and four were nonsignificant (see Table E1 in ESM 1 for all pairwise comparisons). Searches in the Veteran Resources category were not significantly different than the Homelessness category (*OR* = 0.7, 95% CI [0.5, 1.0]). Safety Class had no difference with Firearm Policy (*OR* = 0.7, 95% CI [0.6, 1.2]) or Firearm Storage (*OR* = 0.7, 95% CI [0.6, 1.0]). Finally, Firearm Policy had no difference with Firearm Storage (*OR* = 0.8, 95% CI [0.7, 1.2]).

When comparing overarching domains or categories, ads shown with General Veteran searches were significantly more likely to result in a click compared to searches in the Firearm (*OR* = 3.4, 95% CI [3.2, 3.7]) and Mental Health categories (*OR* = 2.3, 95% CI [2.2, 2.5]). Searches in the Mental Health category were also more likely to have an ad click than Firearm searches (*OR* = 1.5, 95% CI [1.4, 1.6]).

**Figure 3:**
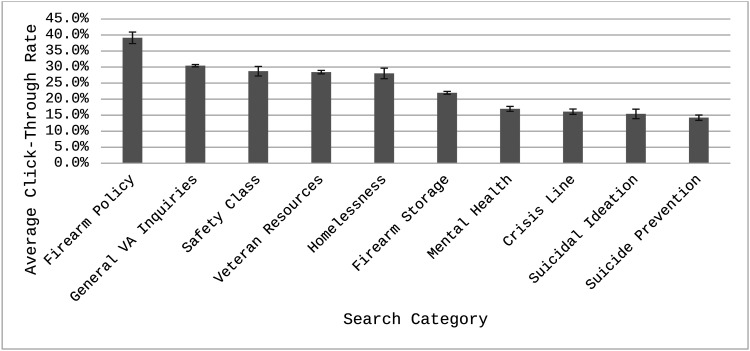
Least squares mean click-through rate (CTR+) across categories. Means are derived from the generalized mixed model predicting CTR. Means are estimated excluding searches with CTRs = 0%. Error bars represent standard errors.

After excluding 0% CTR searches, there was a significant difference in average CTR between categories, *F*(9, 13,073) = 89.4, *p* < .001. Figure 3 shows estimated mean CTR+s from the model. Firearm Policy searches tended to have the highest CTR+ (39.1%, 95% CI [35.6%, 42.7%]), compared to the other categories. Mental Health (17.0%, 95% CI [15.4%, 18.5%]), Crisis Line (16.1%, 95% CI [14.4%, 17.7%]), Suicidal Ideation (15.4%, 95% CI [12.5%, 18.3%]), and Suicide Prevention (14.2%, 95% CI [12.6%, 15.8%]) having the lowest compared to other categories. Of the 45 pairwise comparisons, 33 were statistically significant, and 12 were nonsignificant (see Table E2 in ESM 1 for all pairwise comparisons). Veteran Resources was not significantly different from Safety Class and Homelessness (*p*s = 1.00). Safety Class was not significantly different from Homelessness and General VA Inquiries (*p*s = 1.00). Homelessness was not significantly different from General VA Inquiries (*p* = 1.00). Crisis Line was not significantly different from Mental Health, Suicide Prevention, and Suicidal Ideation (*p*s = 1.00). Suicidal Ideation was not significantly different from Mental Health and Suicide Prevention (*p*s = 1.00). Finally, Mental Health was not significantly different from Suicide Prevention (*p* = .71).

When comparing overarching domains, ads shown with General Veteran searches had a significantly higher average CTR+ (29.7%, 95% CI [29.1%, 30.3%]) than Firearm (23.5%, 95% CI [22.6%, 24.3%]) and Mental Health searches (15.8%, 95% CI [14.9%, 16.6%]; *p*s < .001). Firearm searches had a significantly higher CTR+ than Mental Health searches (*p* < .001).

## Discussion

This study is the first we are aware of to systematically analyze the impact of Google Ads on engagement in a large-scale public health campaign for veteran suicide prevention. Using millions of ad impressions and tens of thousands of Google searches contained in the Keep It Secure paid media campaign to promote lethal means safety in veterans, we identified meaningful patterns in the types of searches made and how they were associated with engagement with the campaign. Several practical insights emerge from our findings.

1. Using the “side-door” approach to keyword targeting: The 10 categories of Google searches linked to engagement in the lethal means safety campaign were wide-ranging and did not necessarily directly relate to firearms or suicide. Indeed, searches related to homelessness, veteran resources, and general inquiries about the VA were most likely to translate into users clicking to the Keep It Secure campaign website. While it is intuitive to bid primarily on keywords directly related to firearms (e.g., gun safes), our data indicate that broader, nonspecific keywords (e.g., VA benefits, eligibility) yielded significantly higher click-through rates. Campaign administrators should allocate a portion of advertising budgets to these types of keywords. This strategy captures a wider audience of veterans who may not be explicitly seeking safety devices but are nonetheless receptive to the messaging when presented with VA information.
2. Tailoring intervention strategy to level of acuity: The low engagement we found associated with searches related to suicidal ideation and crisis lines implies that messaging about secure storage of firearms may be mismatched for users in acute distress. Put another way, when a person searches for terms such as *kill myself*, they likely require immediate intervention rather than preventative storage education. Consequently, campaign managers might consider excluding high-acuity suicide keywords from lethal means safety campaigns and instead reserve these keywords for campaigns directing users to immediate crisis resources, such as the Veterans Crisis Line. This ensures the right resource is matched to right state of mind.
3. Leveraging high-engagement subpopulations: Searches categorized under Homelessness demonstrated the highest likelihood of engagement, with over 52% of searches resulting in a click. This suggests that individuals interested in, or affected by, housing instability, may be particularly vigilant regarding firearm safety and resources. This highlight a unique opportunity to reach the important subpopulation of homeless veterans who face heighted rates of suicide attempt than other veterans (Holliday et al., 2021). Future campaigns could develop tailored ad copy specifically for this demographic – perhaps framing secure storage in the context of personal safety while unhoused or in transitional living.

### Findings in Relation to Prior Studies

Despite the lower *relative* engagement of searches related to suicidal ideation and crisis lines, most of the search categories contained in Keep It Secure’s Google Ad campaign demonstrated robust *absolute* engagement. While it is difficult to know what the normative CTR may be for appropriate comparison, Onie et al. found ranges of 3-6% in their suicide prevention campaigns using Google Ads (Onie et al., 2023, 2024), and CTRs in the range of 3-4% are common for Google Ads in the health/medical and advocacy industries (Irvine, 2024). By comparison, our overall CTR was 18.5%, the average CTR per search was 6.9%, and seven of 10 of our search categories had CTRs >6% (including crisis line), with the only category with poor engagement comparatively was suicidal ideation.

While our study focused on military veterans in the United States, the potential applicability of Google Ads and other search engine ads is much wider in scope. Indeed, a recent study comparing Google Ad campaigns for suicide prevention in three countries found the highest level of engagement in Indonesia, where the campaign was created after a process of codesign and cultural adaption after prior campaigns in the higher-income countries.

Our findings are in line with previous work finding high-risk searches (e.g., “I want to die”) as less likely to lead to clicks on a suicide prevention campaign ad compared to low-risk (e.g., “feeling so alone”) and help-seeking searches (e.g., “suicide help”; Onie et al., 2023). In a men’s mental health campaign, Murphy et al. (2018) also found general, nonspecific searches (e.g., those unrelated to depression and other mental disorders) resulted in the largest number of clicks. Counterintuitively, then, it seems that less targeted keywords and search categories may do best to increase website traffic when using Google Ads. Indeed, most of the searches in our dataset could be considered low-risk, with few relating to mental health issues or firearm safety.

### Limitations and Future Work

There are limitations to the present study that should be mentioned. First, as this was a secondary analysis of the Keep It Secure campaign, data are retrospective and observational in nature. We were provided data after campaign execution, did not have control over the Google Ads messaging, and were unable to perform any experimental manipulations. These limitations preclude any causal inferences, and we cannot adjust for any potential unmeasured confounders. Second, our dataset was limited to website clicks. Future work would benefit from inclusion of additional conversions, which refers to additional behaviors after an individual clicks on an ad (e.g., downloading a firearm safety resource on the website). Future research should identify trackable conversions a priori and evaluate both ad clicks and meaningful behavior changes; in the context of lethal means safety, this could include behaviors such as purchasing a firearm lock or accessing a crisis line from the website. We also utilized one platform (Google Ads) and placement (Google keyword search ads). As such, we may have missed individuals who use other search engines (e.g., Bing, Yahoo). Although Google Ads is far and above the most commonly used platform, public health researchers may wish to consider expanding to more platforms and ad placements to reach a more representative audience. Finally, not all searches were coded, and our restriction to search queries with at least 50 impressions may have led to an inflation of CTR. Future research may benefit from integrating artificial intelligence, including machine learning methods to quantitatively derive categories from qualitative information (Zhang et al., 2020).

### Conclusion

Our study applied a mixed-methods approach to characterize Google Ads used in a national lethal means safety campaign for veterans, and identified 10 distinct categories of Google searches linked to campaign engagement. Our results indicated Google Ads can be an effective tool to reach large numbers of people who are primed to firearms safety messages. We believe our findings suggest actionable steps for a variety of stakeholders – including public health officials, campaign managers, and healthcare organizations such as VA – that will support design of more impactful suicide prevention campaigns.

## Electronic Supplementary Material

The following electronic supplementary material is available at https://doi.org/10.1027/0227-5910/a001057
**ESM 1**. Flow diagram of qualitative analysis process, pairwise comparisons between search categories.

## Notes

**Publication Ethics:** This project was determined to not be human subjects research by the Veterans Affairs Institutional Review Board on May 19, 2022, and it was thus exempt from ethical compliance.

**Authorship:** Alan R. Teo: Conceptualization, methodology, investigation, writing – Original draft, writing – Review and editing, supervision, funding acquisition. Sean P. M. Rice: Methodology, software, validation, formal analysis, data curation, writing – Original draft, writing – Review and editing, visualization. Summer Newell: Methodology, validation, formal analysis, data curation, writing – Original draft, writing – Review and editing, visualization.

**Open Science:** The data that support the findings of this study are available from the corresponding author, Alan R. Teo, upon reasonable request.
