# Supplementary material for: Using Google Search Ads to Promote Lethal Means Safety in Military Veterans: A Mixed-Methods Evaluation of the Keep It Secure Paid Media Campaign
Source: Crisis. 2026 Mar 26;47(3):170–9. doi: 10.1027/0227-5910/a001057 (PMC13094417; doi:10.1027/0227-5910/a001057)
Supplement: Supplementary file 1 [file cri_47_3_170_esm1.pdf]

Electronic Supplementary Material 1 for https://doi.org/10.1027/0227-5910/a001057

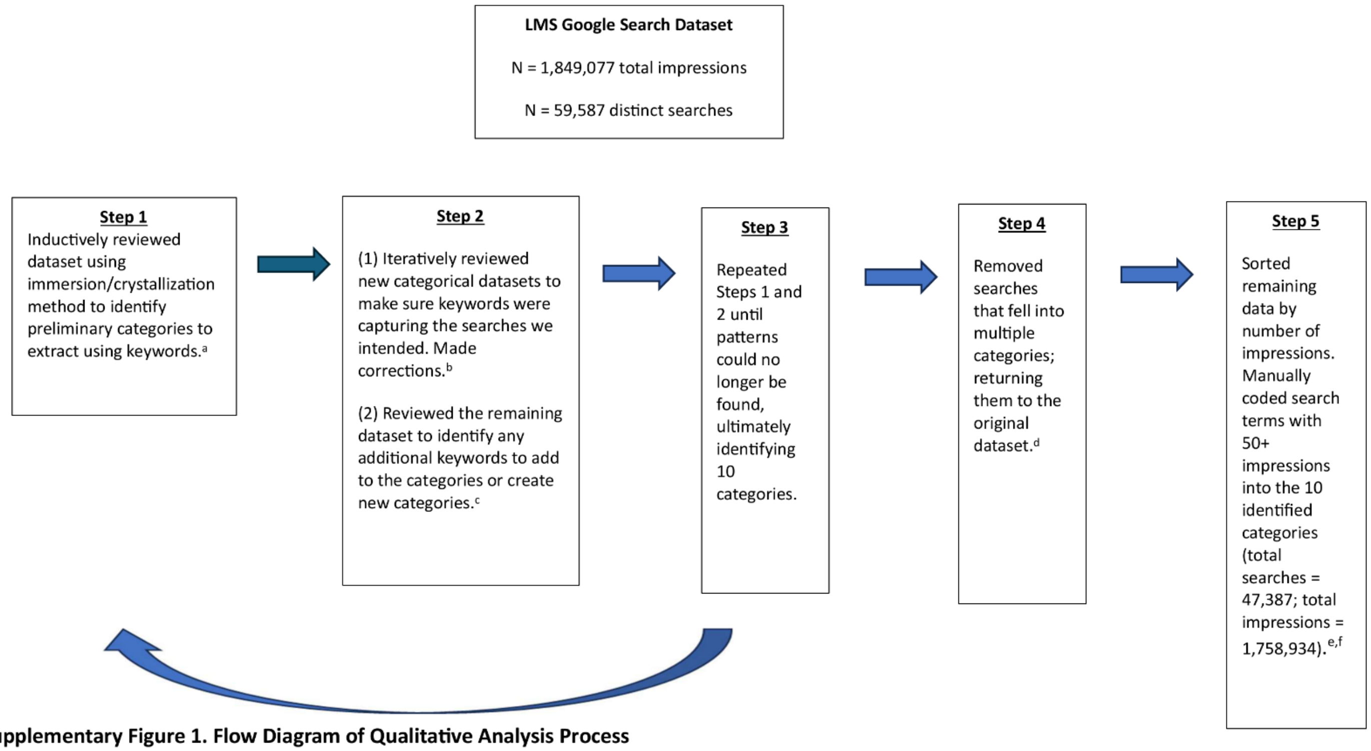

Supplementary Figure 1. Flow Diagram of Qualitative Analysis Process

Note. [a]Two preliminary categories identified: (1) General VA Inquiries Example keywords: VA, veterans affairs; and (2) Gun safety equipment Example keywords: safe, vault, lock. [b]For example: searching for “va” yielded searches with “vault” so we learned that “va” had to be an exact match. [c]For example: It was determined that both singular and plural versions of keywords needed to be included. [d] For Example: “mental health hotline.” [d]Only a handful of the searches with greater than 50 impressions could not be manually coded (e.g., “gunclass mudbaby”, “artificial suicid3”). [f]For searches with keywords from multiple categories (and with greater than 50 impressions) the qualitative analyst manually coded them into the category that the search most closely matched.

© 2026 The Author(s). Distributed as a Hogrefe OpenMind article under the license CC BY 4.0 (https://creativecommons.org/licenses/by/4.0)

Electronic Supplementary Material 1 for https://doi.org/10.1027/0227-5910/a001057

Table E1. Pairwise Comparisons between Search Categories of the Likelihood of a Query Leading to an Ad Click

Search Category	Reference Category	Odds Ratio	95% CI Lower Limit	95% CI Upper Limit
Crisis Line	General VA Inquiries	0.7	0.6	0.8
Crisis Line	Firearm Storage	2.2	1.9	2.6
Crisis Line	Mental Health	1.3	1.1	1.5
Crisis Line	Policy	2.4	1.8	3.3
Crisis Line	Prevention	1.6	1.3	1.9
Crisis Line	Suicidal Ideation	4.0	3.1	5.1
Firearm Storage	General VA Inquiries	0.3	0.3	0.4
Firearm Storage	Suicidal Ideation	1.8	1.4	2.2
Homelessness	Crisis Line	2.3	1.6	3.2
Homelessness	General VA Inquiries	1.6	1.2	2.2
Homelessness	Firearm Storage	5.1	3.7	7.0
Homelessness	Mental Health	2.9	2.0	4.0
Homelessness	Policy	5.6	3.7	8.4
Homelessness	Prevention	3.6	2.6	5.1
Homelessness	Suicidal Ideation	9.1	6.2	13.3
Mental Health	General VA Inquiries	0.6	0.5	0.6

© 2026 The Author(s). Distributed as a Hogrefe OpenMind article under the license CC BY 4.0 (https://creativecommons.org/licenses/by/4.0)

Electronic Supplementary Material 1 for https://doi.org/10.1027/0227-5910/a001057

Mental Health	Firearm Storage	1.8	1.5	2.0
Mental Health	Policy	1.9	1.5	2.6
Mental Health	Prevention	1.3	1.1	1.5
Mental Health	Suicidal Ideation	3.2	2.5	4.0
Policy	General VA Inquiries	0.3	0.2	0.4
Policy	Firearm Storage	0.9	0.7	1.2
Policy	Prevention	0.7	0.5	0.9
Policy	Suicidal Ideation	1.6	1.2	2.3
Prevention	General VA Inquiries	0.4	0.4	0.5
Prevention	Firearm Storage	1.4	1.2	1.6
Prevention	Suicidal Ideation	2.5	2.0	3.2
Safety Class	Crisis Line	0.4	0.3	0.5
Safety Class	General VA Inquiries	0.3	0.2	0.3
Safety Class	Firearm Storage	0.8	0.7	1.0
Safety Class	Homelessness	0.2	0.1	0.2
Safety Class	Mental Health	0.5	0.4	0.6
Safety Class	Policy	0.9	0.6	1.2
Safety Class	Prevention	0.6	0.5	0.7
Safety Class	Suicidal Ideation	1.5	1.1	2.0

© 2026 The Author(s). Distributed as a Hogrefe OpenMind article under the license CC BY 4.0 (https://creativecommons.org/licenses/by/4.0)

Suicidal Ideation	General VA Inquiries	0.2	0.1	0.2
Veteran Resources	Crisis Line	1.7	1.4	2.0
Veteran Resources	General VA Inquiries	1.2	1.1	1.3
Veteran Resources	Firearm Storage	3.8	3.3	4.2
Veteran Resources	Homelessness	0.7	0.5	1.0
Veteran Resources	Mental Health	2.1	1.8	2.5
Veteran Resources	Policy	4.1	3.1	5.4
Veteran Resources	Prevention	2.7	2.3	3.1
Veteran Resources	Safety Class	4.6	3.7	5.9
Veteran Resources	Suicidal Ideation	6.7	5.4	8.5

Note. CI = Confidence Interval. Odds Ratios were calculated by comparing the likelihood of a search leading to a click of the Search Category (first column) relative to the Reference Category (second column). If the Odds Ratio is below 1, then the Search Category had lower odds of having an ad get clicked on compared to the Reference Category. If the Odds Ratio is above 1, then the Search Category had higher odds of having an ad get clicked on compared to the Reference Category. Confidence Intervals were calculated using Bonferroni correction for multiple comparisons. Statistical significance of the difference is inferred when the Confidence Interval does not contain 1.

© 2026 The Author(s). Distributed as a Hogrefe OpenMind article under the license CC BY 4.0 (https://creativecommons.org/licenses/by/4.0)

Electronic Supplementary Material 1 for https://doi.org/10.1027/0227-5910/a001057

Table E2. Pairwise Comparisons between Search Categories of Average Click-Through Rate among Queries with 1 or More Clicks (N = 13,083)

Search Category	Reference Category	Mean Difference	95% CI Lower Limit	95% CI Upper Limit
Veteran Resources	Safety Class	-0.28%	-5.45%	4.89%
Veteran Resources	Homelessness	0.41%	-5.24%	6.06%
Veteran Resources	Crisis Line	12.35%	9.11%	15.59%
Veteran Resources	mental health	11.46%	8.36%	14.56%
Veteran Resources	Policy	-10.71%	-16.82%	-4.61%
Veteran Resources	Prevention	14.21%	10.99%	17.43%
Veteran Resources	Firearm Storage	6.45%	4.15%	8.75%
Veteran Resources	Suicidal Ideation	13.04%	7.92%	18.17%
Veteran Resources	General VA Inquiries	-2.01%	-4.14%	0.12%
Safety Class	Homelessness	0.69%	-6.55%	7.93%
Safety Class	Crisis Line	12.63%	7.06%	18.20%
Safety Class	mental health	11.74%	6.25%	17.23%
Safety Class	Policy	-10.43%	-18.04%	-2.83%
Safety Class	Prevention	14.49%	8.93%	20.05%
Safety Class	Firearm Storage	6.73%	1.65%	11.81%
Safety Class	Suicidal Ideation	13.32%	6.48%	20.16%

© 2026 The Author(s). Distributed as a Hogrefe OpenMind article under the license CC BY 4.0 (https://creativecommons.org/licenses/by/4.0)

Safety Class	General VA Inquiries	-1.73%	-6.74%	3.28%
Homelessness	Crisis Line	11.94%	5.92%	17.96%
Homelessness	mental health	11.05%	5.11%	17.00%
Homelessness	Policy	-11.12%	-19.06%	-3.18%
Homelessness	Prevention	13.81%	7.80%	19.81%
Homelessness	Firearm Storage	6.04%	0.47%	11.61%
Homelessness	Suicidal Ideation	12.63%	5.42%	19.85%
Homelessness	General VA Inquiries	-2.42%	-7.92%	3.09%
Crisis Line	mental health	-0.89%	-4.62%	2.85%
Crisis Line	Policy	-23.06%	-29.51%	-16.61%
Crisis Line	Prevention	1.87%	-1.97%	5.70%
Crisis Line	Firearm Storage	-5.90%	-9.00%	-2.80%
Crisis Line	Suicidal Ideation	0.70%	-4.84%	6.23%
Crisis Line	General VA Inquiries	-14.35%	-17.33%	-11.38%
mental health	Policy	-22.17%	-28.56%	-15.79%
mental health	Prevention	2.75%	-0.96%	6.47%
mental health	Firearm Storage	-5.01%	-7.97%	-2.06%
mental health	Suicidal Ideation	1.58%	-3.87%	7.03%
mental health	General VA Inquiries	-13.47%	-16.29%	-10.65%

© 2026 The Author(s). Distributed as a Hogrefe OpenMind article under the license CC BY 4.0 (https://creativecommons.org/licenses/by/4.0)

Electronic Supplementary Material 1 for https://doi.org/10.1027/0227-5910/a001057

Policy	Prevention	24.93%	18.48%	31.37%
Policy	Firearm Storage	17.16%	11.13%	23.20%
Policy	Suicidal Ideation	23.76%	16.18%	31.33%
Policy	General VA Inquiries	8.71%	2.73%	14.68%
Prevention	Firearm Storage	-7.77%	-10.85%	-4.68%
Prevention	Suicidal Ideation	-1.17%	-6.69%	4.35%
Prevention	General VA Inquiries	-16.22%	-19.18%	-13.27%
Firearm Storage	Suicidal Ideation	6.60%	1.56%	11.63%
Firearm Storage	General VA Inquiries	-8.46%	-10.37%	-6.55%
Suicidal Ideation	General VA Inquiries	-15.05%	-20.01%	-10.09%

Note. CI = Confidence Interval. Mean Differences were calculated by comparing the average Click-Through Rate (CTR) per search query of the Search Category (first column) relative to the Reference Category (second column). If the Mean Difference is negative, then the Search Category had a lower CTR compared to the Reference Category. If the Mean Difference is positive, then the Search Category had a higher CTR compared to the Reference Category. Confidence Intervals were calculated using Bonferroni correction for multiple comparisons. Statistical significance of the difference is inferred when the Confidence Interval does not contain 0. Averages were calculated after excluding search queries with a CTR of 0 to account for the zero-inflated nature of the data.

© 2026 The Author(s). Distributed as a Hogrefe OpenMind article under the license CC BY 4.0 (https://creativecommons.org/licenses/by/4.0)
